# Unemployment and work disability in individuals with chronic fatigue syndrome/myalgic encephalomyelitis: a community-based cross-sectional study from Spain

**DOI:** 10.1186/s12889-019-7225-z

**Published:** 2019-06-28

**Authors:** Jesús Castro-Marrero, Mónica Faro, María Cleofé Zaragozá, Luisa Aliste, Tomás Fernández de Sevilla, José Alegre

**Affiliations:** 1 CFS/ME Unit, Vall d’Hebron University Hospital Research Institute, Universitat Autónoma de Barcelona, Passeig de Vall d’Hebron 119-129, E-08035 Barcelona, Spain; 2 EAP CAP Terrassa Nord, Consorcio Sanitario de Terrassa, Barcelona, Spain; 3 Clinical Research Department, Laboratorios Viñas, Barcelona, Spain

**Keywords:** Chronic fatigue syndrome, Myalgic encephalomyelitis, Work disability, Unemployment, Quality of life, Comorbidity

## Abstract

**Background:**

Few reports have examined the association between unemployment and work disability in Chronic Fatigue Syndrome/Myalgic Encephalomyelitis (CFS/ME). This study explored the key determinants of work disability in a CFS/ME cohort.

**Methods:**

A community-based prospective study included 1086 CFS/ME patients aged 18–65 years. Demographic and clinical characteristics and outcome measures were recorded. Multiple linear regression analysis was performed to identify key risk indicators of work disability.

**Results:**

Four hundred and fifty patients with CFS/ME were employed (41.4%) and 636 were unemployed (58.6%). Older age at pain onset (OR: 1.44; 95% CI: 1. 12–1.84, autonomic dysfunction (OR: 2.21; 95% CI: 1.71–2.87), neurological symptom (OR: 1.66; 95% CI: 1. 30–2.13) and higher scores for fatigue (OR: 2.61; 95% CI: 2.01–3.39), pain (OR: 2.09; 95% CI: 1.47–2.97), depression (OR: 1.98; 95% CI: 1. 20–3.26), psychopathology (OR: 1.98; 95% CI: 1.51–2.61) and sleep dysfunction (OR: 1.47; 95% CI: 1. 14–1.90) were all associated with a higher risk of work disability due to illness.

**Conclusions:**

Using an explanatory approach, our findings suggest that unemployment is consistently associated with an increased risk of work disability due to CFS/ME, although further more rigorous research is now needed to help in targeting interventions at the workplace.

## Key points

1. This study examined the effect of unemployment on work disability due to cardinal symptoms and comorbidity in individuals with CFS/ME.
2. Work disability is common in adult patients with CFS/ME. Further investigations should aim to define the phenotype of patients who would benefit from specific interventions, and should develop clinically relevant objective measures.
3. The results may provide useful guidance for the design of rehabilitation strategies to counter work disability, raise quality of life, and reduce future productivity losses in those CFS/ME individuals of Europe.
4. Targeting chronic fatigue-associated symptoms through the design of public health care and occupational policies may help to reduce the burden of CFS/ME in Europe.

## Background

Chronic fatigue syndrome/myalgic encephalomyelitis (CFS/ME) is a serious, complex, multisystem, neuroimmune condition with a variety of clinical presentations that affect millions of people all over the world. CFS/ME is a debilitating illness that substantially impairs professional, recreational, social, and educational activities. Despite substantial efforts by researchers to unravel the pathomechanisms that underlying to CFS/ME, no known cause has been identified, no diagnostic tests have been established, and no FDA-approved drugs are available for effective treatment [1]. CFS/ME is characterized by debilitating chronic fatigue, widespread pain, post-exertional malaise, unrefreshing sleep, cognitive impairments and autonomic dysfunction, which affect daily physical and work activity. Often patients do not receive appropriate treatment and are ineligible for the disability benefits awarded to other chronic diseases, in spite of the significant social burden of the condition [2]. So far, the influence of employment status on work disability in CFS/ME individuals in Spain has not been fully investigated. Nor have any epidemiological studies have been conducted in this country, though elsewhere the estimated prevalence ranges between 0.07 and 2.6% of the general population [3–5]. The 1994 CDC/Fukuda definition for CFS/ME requires that individuals experience 6 months of fatigue not alleviated by rest, is of new or definite onset and is accompanied by at least four of eight core symptoms present for at least six consecutive months [6]. A new definition of CFS/ME excluding psychiatric cases was proposed in the Canadian consensus criteria [7]; this useful complement to the 1994 CDC/Fukuda case definition. Diagnosis is difficult for physicians who are not familiar with the illness. For this reason, many CFS/ME patients may initially be misdiagnosed with other fatiguing illnesses (the opposite is also true; many patients with other complex fatiguing-related conditions have been misdiagnosed with CFS and/or ME). However, by following standard medical procedures – taking a careful/thorough history, ruling out similar fatigue-related illnesses, noting signs and symptoms typical of the condition, any medications the patient is currently taking, any other factors that might influence the severity or persistence of the fatigue, and ordering tests that are usually abnormal in CFS/ME patients, any physician can diagnose the disease. CFS/ME is also associated with comorbid health conditions. These comorbidities very in terms of prevalence and severity, but are higher than in patients without CFS/ME [8, 9]. The study of CFS/ME includes assessment of fatigue, pain, anxiety-depression, psychopathological symptoms, sleep dysfunction and quality of life (QoL) using validated self-administered questionnaires. QoL in CFS/ME individuals is significantly worse than in other chronic diseases [10]. CFS/ME is associated with significant alterations in work and family life; and impact their economic wellbeing [11–13]. To date, few studies have attempted to explore the effects of work disability on employment status in CFS/ME, and most of the studies carried out were underpowered or were conducted in highly selected populations [14, 15]. The present study aims to identify key factors of work disability in a large Spanish CFS/ME sample in order to design interventions for overcoming them.

## Methods

### Study setting and participants

This large community-based prospective cohort study included 1086 potentially eligible patients who fulfilled the 1994 CDC/Fukuda definition and 2003 Canadian Consensus Criteria for CFS and ME, respectively [6, 7]. Only CFS/ME subjects of working-age (i.e., adults aged 18–65 years) and recruited at a single outpatient tertiary-referral center (CFS/ME Unit, Vall d’Hebron University Hospital, Barcelona, Spain) over a 10 year follow-up period beginning in January 2008 were included. Data source for baseline information was provided from the Spanish National Health System (SNHS) based on the local CFS/ME clinical data register. Exclusion criteria were psychiatric conditions, cardiovascular, hematological, infectious, endocrine and metabolic disorders, autoimmune conditions, pregnancy or breastfeeding, current drug/substance abuse, and smoking. All procedures performed were reviewed and approved by the Human Research Ethics Committee of the Vall d’Hebron University Hospital, Barcelona, Spain. Participants were fully informed of the study procedures, and provided written informed consent prior to study initiation. The study was conducted in accordance with the Declaration of Helsinki, no extra procedures were performed, and data were analyzed in an irreversibly anonymized fashion.

### Study variables

#### Covariates

We recorded demographic and clinical variables such as age, gender, and comorbid health conditions. We also assessed the characteristics of fatigue and pain (apparent cause, age at onset, course and time of evolution). Illness duration, marital status, type of profession and educational level were also recorded as is categorized in Table 1. According to the definition of SNHS through the Ministry of Employment and Social Security, participants who self-reported that they had been out of work for 1 year or more were classified as unemployed. Those who indicated they were “employed for wages” or “self-employed” were classified as employed.

**Table 1:** Baseline socio-demographic and clinical characteristics based on employment status of the study population (*n* = 1086)

	Employed (*n* = 450)	Unemployed (*n* = 636)	*P-*value^b^
Gender			0.959
Female	405 (90.0)	573 (90.1)	
Male	45 (10.0)	63 (9.9)	
Age group, years			0.004^**^
≤ 40	123 (27.3)	130 (20.4)	
41–50	182 (40.4)	246 (38.7)	
> 50	145 (32.2)	260 (40.9)	
Age at onset, years			0.002^**^
Fatigue	36 ± 10.3	37.8 ± 9.5	
Pain	36.6 ± 10.2	38.4 ± 9.6	
Illness duration, years	9 ± 7.6	10.5 ± 8.6	0.839
Comorbid conditions^a^			
Fibromyalgia	207 (46.0)	394 (62.0)	< 0.001^***^
Degenerative vertebral disease	220 (49.0)	376 (59.3)	0.001^***^
Epicondylitis	187 (41.6)	310 (48.9)	0.018^*^
Ligamentous hyperlaxity	130 (28.9)	235 (37.1)	0.005^**^
Multiple chemical hypersensitivity	135 (30.1)	230 (36.2)	0.035^**^
Carpal tunnel syndrome	62 (13.8)	122 (19.2)	0.018^*^
Marital status			0.028^**^
Married	272 (60.6)	428 (67.4)	
Single	107 (23.8)	107 (16.9)	
Separated/divorced	60 (13.4)	90 (14.2)	
Widow	10 (2.2)	10 (1.6)	
Profession			< 0.001^***^
Unskilled work	213 (47.3)	262 (41.2)	
Skilled work	105 (23.3)	179 (28.1)	
Administrative/office work	92 (20.4)	111 (17.5)	
Education/teaching	14 (3.1)	15 (2.4)	
Self-employed	7 (1.6)	7 (1.1)	
Liberal profession	16 (3.6)	23 (3.6)	
Arts and crafts	1 (0.2)	5 (0.8)	
Student	2 (0.4)	1 (0.2)	
Housewife	0 (0)	33 (5.2)	
Educational level			0.506
Elementary (primary) school	69 (15.3)	109 (17.1)	
Graduate	66 (14.7)	105 (16.5)	
High school/vocational training	188 (41.8)	248 (39.0)	
University	126 (28.0)	169 (26.6)	
Literate	1 (0.2)	5 (0.8)	

Data are given as numbers of cases (percentages) unless otherwise indicated

^*^Significance at 0.05

^**^Significance at 0.01

^***^Significance at 0.001

^a^ Because participants may report more than one comorbid illness, column numbers do not add up to the total sample. ^b^ Data analysis was performed using Chi-square test for categorical variables

#### Confounders

As potential confounders, we included gender, age, marital status, education level, neurocognitive symptoms and muscular skeletal pain including the pain onset at baseline which were examined in relation to their effects on the results. Information on gender, age, marital status’ and ‘vocational education’ was obtained from the registry. Applying the ISCED classification, all persons were grouped into married, single, separated/divorced or widow for marital status and primary, secondary and tertiary education, respectively. Because musculoskeletal disorders and related pain are known causes of disability, we used self-reported VAS ‘chronic high or low back pain’ as indicator for co-morbid conditions. Persons were considered exposed when they perceived pain ‘all the time’, and persons were considered unexposed when they perceived ‘less pain’.

#### Measures

All eligible participants completed the following self-reported outcome questionnaires.

#### Visual analog scale (VAS)

Patients were asked to self-report their pain/fatigue symptom intensity on a visual analog scale (VAS), a continuous single-item scale (one-dimensional measure for pain/fatigue intensity) comprising a 10 cm-length line ranging from “no pain/fatigue” (score of 0 cm) to “pain/fatigue as bad as it could be” or “worst imaginable pain/fatigue” (score of 10 cm). Patients mark the VAS line at the point that represents their “current” symptom intensity “in the last 24 hours”. Higher scores indicated greater pain/fatigue intensity [16].

#### Fatigue impact Scale-40 (FIS-40)

The FIS-40 assesses the effects of fatigue on QoL and examines the perception of functional limitations due to fatigue in the last month. It reflects the perceived impact of fatigue in three subscales: cognitive functioning (10 items), physical functioning (10 items), and psychosocial functioning (20 items). Each item is scored from 1 (absence of problem) to 4 (maximum problem). The maximum score is 160 points. Scores over 120 points were considered as clinically severe fatigue symptoms, and a score of 120 points or below was defined as mild/moderate symptoms. Internal consistency was high for overall scores and the three subscales (Cronbach’s α ≥ 0.87). Test–retest reliability was high (0.72–0.83), as was convergent validity for CFS/ME individuals and healthy controls [17].

#### Daily-fatigue impact Scale-8 (D-FIS-8)

The D-FIS-8 was developed from the FIS-40 questionnaire to measure the response to daily changes in fatigue intensity. It comprises seven items with Likert type responses with seven possibilities of increasing intensity, scoring from 1 to 7. The total score is the sum of all the items. This 8-item D-FIS has demonstrated good correlations with CFS/ME symptom ratings and other general health ratings [18].

#### Short form 36-item health survey (SF-36)

This generic self-report scale is used to assess health-related quality of life (HR-QOL), measuring physical and mental functioning within the context of an individual’s health status. It comprises 36 questions which explore eight dimensions of the state of health: physical functioning (limitation of physical activities of daily life), physical role functioning (interference at work), bodily pain, general health perception, vitality, social functioning (interference in habitual social life), emotional role (interference in work due to emotional problems) and mental health (depression, anxiety, self-control and general well-being). The SF-36 produces two summary components, one physical and one mental, each one generated by combining the scores of each dimension. SF-36 scores range from 0 to 100, with scores below 50 indicating a more disabling effect of the individual’s health on his/her functioning [19].

#### Symptom CheckList-90-revised (SCL-90-R)

This inventory evaluates a wide range of self-reported psychological problems and psychopathological symptoms. Each of the 90 items on the questionnaire is assessed on a five-point rating scale (0–4) and is evaluated and interpreted based on nine primary symptom domains and three global psychological distress indices. Primary symptom dimensions are: somatization, obsessive-compulsive, interpersonal sensitivity, depression, anxiety, hostility, phobic anxiety, paranoid ideation and psychotics, and the global indices are: global severity index, positive symptom distress index, and overall positive symptoms. Total scores of 65 or over indicate people at mild/moderate symptom risk, and scores of 80 or more indicate the presence of severe psychopathological symptoms. The questionnaire performs an initial evaluation of patients as an objective assessment of the symptom, to measure the patient’s progress (before, during and after treatment) and treatment outcomes; in clinical trials, it is used also to measure changes in symptoms such as depression and anxiety. More than 1000 studies have been conducted demonstrating the reliability, validity, and utility of the instrument [20].

### Hospital anxiety and depression scale (HADS)

The HADS is a 14-item scale including two subscales, anxiety and depression, each one comprising seven statements, in which the frequency or intensity of baseline anxiety (HADS-A) and depression (HADS-D) symptoms is measured on a four-point Likert scale (from 0 to 3). The total score on each subscale is obtained by adding together the items, and thus ranges from 0 to 21. A score ≥ 8 on HADS indicates the presence of clinically significant anxiety/depression symptoms. Scores of 0–7 indicate the absence of significant morbidity. The severity of anxiety/depression symptoms was classified as “mild/moderate” (HADS ≥8 and ≤ 14), or “severe” (HADS ≥15 and ≤ 21) [21].

### Pittsburgh sleep quality index (PSQI)

The PSQI is considered one of the best scales for quantitative assessment of the quality of sleep in a wide variety of conditions. It comprises 24 questions, of which 19 must be answered by the subject and the remaining five by the roommate (if applicable). After correction, seven scores are obtained that provide information on various aspects of sleep quality (subjective sleep quality, sleep latency, sleep duration, habitual sleep efficiency, sleep disturbances, use of sleeping medication, and daytime dysfunction). Each component is scored from 0 (no problems) to 3 points (serious sleep problems). The global PSQI score ranges from 0 to 21 points, with a global PSQI score ≥ 5 indicating poor sleep quality or ‘poor sleepers’ [22].

### Statistical analysis

The descriptive variables analyzed were employment status at the time of diagnosis (employed, and unemployed due to sick leave or disability) as dependent variable, and demographic and clinical variables (age, sex, comorbid health conditions, marital status, profession, and educational level) as independent variables. The sample was described by means of relative and absolute frequencies, dispersion and central tendency: mean, median, standard deviation, minimum, maximum and the valid number of cases. Sample homogeneity was checked by the Kolmogorov-Smirnov test to ensure normal distribution and the Levene’s test to verify the homogeneity of variances. In order to facilitate the analysis, the continuous variables (age at onset of pain and fatigue, VAS for fatigue and pain, muscular, cognitive, neurological, autonomic and immune symptoms, and all self-reported outcome measures) were categorized according to their median values. The association between categorical variables was analyzed using the Pearson correlation coefficient (χ^2^-test). The comparisons of means between employed and unemployed participants for continuous variables were analyzed using the non-parametric Mann-Whitney U test for independent samples. To determine risk indicators associated with work disability, a univariate logistic regression analysis was performed for each of the variables. Multivariate analysis was performed using backward logistic regression analysis, keeping age and gender as dependent variables. The type I error was set at 5% based on a standardized normal deviation (*p* <  0.05) with a 95% CI. All statistical analyses were performed using SPSS v21.0 software.

## Results

### Participant characteristics

Participants’ socio-demographic and clinical characteristics and employment status is summarized in Table 1**.** In all, 450 participants were in employment (41.4%) and 636 unemployed (58.6%), of whom 418 (66%) were on sick leave and 218 (34%) had an illness-related disability. The majority of patients were women (90% of those in employment and 90.1% of those unemployed) without any significant differences between the two groups (Table 1). As shown in Table 1, 27.3% of those in employment were aged ≤40 years compared to 20.4% of the unemployed, and 32.2% of those in employment were over 50 years compared to 40.9% of the unemployed (*p* = 0.004). Regarding professional activity, 47.3% of those in employment and 41.2% of the unemployed were unskilled, while 23.3% of those in employment were skilled workers and 28.1% of the unemployed (*p* <  0.001). In patients in employment, the age of onset of fatigue and pain was lower than in the unemployed (mean age ± SD (years): 36.0 ± 10.3 vs. 37.8 ± 9.5 and 36.6 ± 10.2 vs. 38.4 ± 9.5; *p* = 0.002 for both) respectively. Table 1 also shows that the unemployed patients presented more comorbidities. The comorbidities that differed the most between the groups were fibromyalgia (*p* <  0.001), degenerative vertebral disease (*p* = 0.001), epicondylitis (*p* = 0.018), ligamentous hyperlaxity (*p* = 0.005) and carpal tunnel syndrome (*p* = 0.018). There were no significant differences between the groups in terms of family or personal history of illness, apparent cause, or form of onset of fatigue and pain (*data not shown*).

### Influence of self-reported symptom clusters and work status among participants

Regarding the self-reported symptom clusters, unemployed patients had more muscular, cognitive, neurological, autonomic and immunological symptoms than those in employment (*p* <  0.001 across all groups) (Table 2).

**Table 2:** Data on self-reported symptom clusters in the sample (*n* = 1086)

Symptoms	Employed (*n* = 450)	Unemployed (*n* = 636)	*p-*value^a^
Muscular			
Generalized chronic pain	380 (84.4)	583 (91.7)	< 0.001^***^
Muscle weakness	439 (97.6)	619 (97.3)	0.815
Post-exertional malaise	441 (98.0)	630 (99.1)	0.142
Difficulty performing fine movements due to pain	389 (86.4)	553 (86.9)	0.809
Marked muscle contractures	396 (88.0)	567 (89.2)	0.555
Myoclonic	156 (34.7)	264 (41.6)	0.023^*^
Falls due to loss of tone	74 (16.4)	159 (25.0)	0.001^***^
Cognitive			
Concentration impairments	424 (94.2)	616 (96.9)	0.034^*^
Alterations in short-term memory consolidation	410 (91.1)	610 (95.9)	0.001^***^
Alterations during task planning	355 (78.9)	577 (90.7)	< 0.001^***^
Difficulty with calculation	385 (85.6)	584 (91.8)	0.001^***^
Difficulty redding	402 (89.3)	594 (93.4)	0.017^*^
Confusion and forgetfulness	361 (80.2)	560 (88.1)	< 0.001^***^
Temporal-spatial disorientation	282 (62.7)	481 (75.6)	< 0.001^***^
Episodes of nominal aphasia	382 (84.9)	565 (88.8)	0.055
Auditory and visual agnosia	133 (29.6)	257 (40.4)	< 0.001^***^
Neurological			
Ataxia and/or dissymmetry	331 (73.6)	514 (80.8)	0.005^**^
Sensory hypersensitivity	403 (89.6)	582 (91.5)	0.275
Visual alterations	290 (64.4)	472 (74.2)	0.001^***^
Motor incoordination, with or without falls	339 (75.3)	511 (80.3)	0.048^*^
Autonomic			
Dizziness or cephalic instability	356 (79.1)	552 (86.8)	0.001^***^
Vertigo	322 (71.6)	496 (78.0)	< 0.001^***^
Orthostatic hypotension	342 (76.0)	512 (80.5)	0.009^**^
Lipothymia	80 (17.8)	171 (26.9)	0.017^*^
Syncope	48 (10.7)	98 (15.4)	0.015^*^
Frequent palpitations	313 (69.6)	495 (77.8)	0.074
Tremor	171 (38.0)	314 (49.4)	< 0.001^***^
Profuse sweating	285 (63.3)	451 (70.9)	0.024^*^
Altered bowel habits	296 (65.8)	467 (73.4)	0.002^**^
Alterations in urination	224 (49.8)	395 (62.1)	< 0.001^***^
Reduced libido/anorgasmia/impotence	314 (69.8)	489 (76.9)	0.008^**^
Difficulties in visual accommodation	321 (71.3)	494 (77.7)	0.007^**^
Inmunological			
Recurrent low-fever	326 (72.4)	473 (74.4)	0.478
Recurrent odynophagia	306 (68.0)	477 (75.0)	0.060
Painful lymph nodes	236 (52.4)	389 (61.2)	0.061
Raynaud’s phenomenon	125 (27.8)	225 (35.4)	0.552
Generalized morning numbness	343 (76.2)	521 (81.9)	0.743
Migratory arthralgias	376 (83.6)	547 (86.0)	0.996
Allergy to multiple medications	95 (21.1)	168 (26.4)	0.011^*^
Food intolerance	41 (9.1)	78 (12.3)	0.004^**^
Allergy to multiple metals	51 (11.3)	110 (17.3)	0.008^**^
History of sinusitis	30 (6.7)	63 (9.9)	0.022^*^
Facial swelling	19 (4.2)	44 (6.9)	0.265
Mouth ulcers	238 (52.9)	348 (54.7)	0.044^*^
Herpes	226 (50.2)	313 (49.2)	0.101
Candidiasis	172 (38.2)	243 (38.2)	0.006^**^

Data are expressed as numbers of cases (percentages) among sample unless otherwise indicated

^*^Significance at 0.05

^**^Significance at 0.01

^***^Significance at 0.001

^a^ Data analysis was performed using Chi-square test for categorical variables

### Potential predictors assessed by self-reported measures and work status using the univariate model

Table 3 summarizes the mean scores for outcome measures according to employment status among participants. In the pain and fatigue VAS, unemployed patients obtained higher scores (*p* = 0.002). Unemployed patients obtained significantly higher scores on the two fatigue impact questionnaires, D-FIS-8 and FIS-40 (in the latter, on the overall score and on all three items, *p* <  0.001). Unemployed patients reported greater fatigue intensity according to the fatigue intensity scale (*p* <  0.001) (Table 3).

**Table 3:** Mean scores for outcome measures as predictor variables of employment status among participants (*n* = 1086)

Measures	Employed (*n* = 450)	Unemployed (*n* = 636)	*p-*value^a^
VAS-pain	7.3 ± 1.9	7.6 ± 1.8	< 0.001^***^
VAS-fatigue	8.1 ± 1.0	8.4 ± 1.2	< 0.001^***^
FIQ	57.0 ± 7.4	59.6 ± 6.2	< 0.001^***^
FIS-40			
Global score	123.1 ± 22.7	133.3 ± 19.8	< 0.001^***^
Physical	34.2 ± 4.9	36.1 ± 3.7	< 0.001^***^
Cognitive	30.3 ± 7.0	32.7 ± 6.9	< 0.001^***^
Psychosocial	58.4 ± 13.2	64.6 ± 11.2	< 0.001^***^
D-FIS-8	23.3 ± 5.0	25.8 ± 4.4	< 0.001^***^
SCL-90-R			
Global severity index	1.6 ± 0.8	1.9 ± 0.8	< 0.001^***^
Positive symptom distress index	2.4 ± 0.5	2.6 ± 0.5	< 0.001^***^
Global positive symptom	59.5 ± 17.9	62.9 ± 17.6	0.002^**^
Somatization	2.4 ± 0.8	2.7 ± 0.8	< 0.001^***^
Obsessive-compulsive	2.4 ± 0.9	2.6 ± 0.9	0.001^***^
Interpersonal sensitivity	1.4 ± 1.0	1.5 ± 1.0	0.040^*^
Depression	2.0 ± 0.9	2.3 ± 0.9	< 0.001^***^
Anxiety	1.5 ± 1.0	1.7 ± 1.0	< 0.001^***^
Hostility	1.2 ± 1.0	1.2 ± 1.0	0.400
Phobic anxiety	1.1 ± 1.0	1.5 ± 1.1	< 0.001^***^
Paranoid ideation	1.1 ± 1.0	1.2 ± 1.0	0.544
Psychoticism	1.0 ± 0.8	1.1 ± 0.8	0.007^**^
HADS			
Global score	20.5 ± 4.6	22.6 ± 4.5	0.002^**^
Anxiety	10.7 ± 4.8	11.1 ± 4.7	0.209
Depression	9.8 ± 4.8	11.3 ± 4.6	< 0.001^***^
PSQI			
Global score	12.7 ± 4.4	14.0 ± 4.2	< 0.001^***^
Subjective sleep quality	2.0 ± 0.9	2.2 ± 0.9	0.001^**^
Sleep latency	1.9 ± 1.0	2.1 ± 0.9	0.002^**^
Sleep duration	1.7 ± 0.9	1.6 ± 1.0	0.037^*^
Habitual sleep efficiency	1.4 ± 1.2	1.7 ± 1.2	< 0.001^***^
Sleep disturbances	2.0 ± 0.7	2.2 ± 0.6	< 0.001^***^
Use of sleeping medication	1.6 ± 1.4	2.0 ± 1.4	< 0.001^***^
Daytime dysfunction	2.2 ± 0.9	2.2 ± 0.9	0.649
SF-36			
Physical functioning	40.3 ± 21	28.5 ± 18.9	< 0.001^***^
Physical role	8.8 ± 20.8	3.1 ± 13.1	< 0.001^***^
Bodily pain	24.5 ± 19.2	17.3 ± 17	< 0.001^***^
General health	26.3 ± 15.9	21.3 ± 14.4	< 0.001^***^
Vitality	16.9 ± 14.1	13.2 ± 14.1	< 0.001^***^
Social role functioning	37.6 ± 24.4	26.8 ± 22.2	< 0.001^***^
Emotional role functioning	47.0 ± 45.5	38.3 ± 45.1	0.001^***^
Mental health	44.7 ± 20.7	41.6 ± 21.7	0.012^*^
Physical component	28.0 ± 7.4	24.7 ± 6.4	< 0.001^***^
Mental component	35.1 ± 12.7	32.9 ± 13.1	0.004^**^

Data are given as mean ± standard deviation (SD) in the sample

*Abbreviations*: *VAS* Visual analogue scale, *FIQ* Fatigue intensity questionnaire, *FIS* Fatigue impact scale, *SCL-90-R* Symptom checklist-90-revised, *HADS* Hospital anxiety and depression scale, *PSQI* Pittsburgh sleep questionnaire index, *SF-36* Short form 36-item health survey

^*^Significance at 0.05

^**^Significance at 0.01

^***^Significance at 0.001

^a^Data analysis using the Mann-Whitney *U* test for independent samples

Quality of life was affected in all patients, with mean overall scores below 45 on all SF-36 questionnaire items. All items and physical and mental total scores indicated significantly worse QoL in the unemployed patients. The lowest scores were recorded for the physical role (3.1 ± 13.1 in the unemployed and 8.8 ± 20.8 in those in employment), vitality (13.2 ± 14.1 in the unemployed and 16.9 ± 14.1 in those in employment) and bodily pain (17.3 ± 17.0 in unemployed and 24.5 ± 19.2 in those in employment). All items showed significant differences with *p* <  0.001, except for emotional role (*p* = 0.001) and mental health (*p* = 0.012). Analyzing the total physical and mental health scores, physical health was more affected than mental health (*p* <  0.001 vs. *p* = 0.004) (Table 3). On the SCL-90-R questionnaire, unemployed patients scored significantly worse on the three global indexes. Of the nine items, with the exceptions of hostility and paranoid ideation, the symptoms were significantly more severe in the unemployed patients (Table 3). In the HADS questionnaire, the mean scores on the anxiety subscale were 10.7 ± 4.8 in the employed patients and 11.1 ± 4.7 in the unemployed patients (*p* = 0.209); on the depression subscale, mean scores were 9.8 ± 4.8 in patients in employment and 11.3 ± 4.6 in the unemployed (*p* <  0.001).

The PSQI showed that both employed and unemployed patients with CFS/ME had poor sleep quality, but that the unemployed individuals slept significantly worse (*p* <  0.001, Table 3).

### Association between potential self-reported variables and work status in the multivariate model

Table 4 depicts the (clinical) risk factors that were significantly associated with work disability in the sample. In the univariate model, female age was found a differential factor, with patients over 50 at diagnosis more likely to be unemployed (OR = 2.21, 95% CI: 1.41–3.46; *p* <  0.001). Higher age at fatigue and pain onset was associated with work inactivity but the differences were not statistically significant. Presenting more muscular, cognitive, neurological, autonomic and immunological symptoms was a risk factor for unemployment. Regarding comorbidities, FM and degenerative vertebral disease were risk factors for being unemployed. With regard to the measures, higher scores on the pain VAS, higher scores on the four subscales that assess fatigue (FIS-40) and more psychopathological symptoms on the SCL-90-R were predictive risk factors for unemployment. On the HADS, scores possibly indicating symptoms were also a risk factor, as was worse sleep quality assessed on the PSQI questionnaire. A higher physical and/or mental QoL on the SF-36 was a protective factor against being unemployed (Table 4). On the other hand, the multivariate model identified age over 50 years, more autonomic symptoms, high scores on the fatigue VAS and the D-FIS-8 and doubtful scores on the HADS as risk predictors of work disability. Higher physical and mental QoL (assessed by SF-36) were protective factors against being unemployed.

**Table 4:** Multiple lineal regression analysis for self-reported outcome measures with sex and age as dependent variables in the sample (*n* = 1086)

		UNIVARIATE		MULTIVARIATE	
Variable	*n*	OR (95% CI)	*p*-value^a^	OR (95% CI)	*p-*value^a^
Gender					
Female	978	1.01 (0.68–1.51)	0.959	0.97 (0.56–1.67)	0.906
Male	108	1		1	
Age range, years					
≤ 40	253	1		1	
41–50	428	1.28 (0.94–1.75)	0.123	1.59 (1.04–2.42)	0.031^*^
> 50	405	1.70 (1. 23–2.34)	0.001^***^	2.21 (1.41–3.46)	< 0.001^***^
Age of pain onset					
Early, ≤ 3 yrs	532	1		1	
Late, > 3 yrs	520	1.44 (1. 12–1.84)	0.004^**^	1.49 (0.75–2.95)	0.254
VAS (fatigue/pain)					
Lower	645	1		1	
Higher	441	2.24 (1.73–2.89)	< 0.001^***^	2.09 (1.47–2.97)	< 0.001^***^
Cognitive symptoms					
Severe	846	1		1	
Mild/moderate	61	1.66 (1. 30–2.13)	< 0.001^***^	0.28 (0.05–1.64)	0.159
No symptoms	179	0.76 (0.47–1.23)	0.077^**^	0.60 (0. 37-0.96)	0.033^*^
Neurological symptoms					
Severe	606	1		1	
Mild/moderate	451	1.66 (1. 30–2.13)	< 0.001^***^	1.37 (0.92–2.04)	0.117
No symptoms	29	0.82 (0.51–1.23)	0.214	11.02 (2. 24–54.29)	0.003^**^
Autonomic dysfunction					
Severe	412	1		1	
Mild/moderate	161	2.21 (1.71–2.87)	< 0.001^***^	0.22 (0.03–1.51)	0.122
No symptoms	513	0.81 (0. 37–1.76)	0.093	0.57 (0. 38–0.83)	0.004^**^
Fibromyalgia					
No	464	1		1	
Yes	601	1.92 (1.50–2.45)	< 0.001^***^	1.27 (0.87–1.83)	0.214
Ligamentous Hyperlaxity					
No	719	1		1	
Yes	365	1.45 (1. 12–1.88)	0.005^**^	1.36 (0.94–1.97)	0.100
SF-36 (physical health)					
Lower	542	1		1	
Higher	541	0.48 (0. 38–0.62)	< 0.001^***^	0.45 (0. 31–0.66)	< 0.001^***^
SF-36 (mental health)					
Lower	542	1		1	
Higher	541	0.66 (0.52–0.84)	< 0.001^***^	0.50 (0. 33–0.75)	< 0.001^***^
HADS					
No symptoms	250	1		1	
Mild/moderate	583	1.82 (1. 35–2.45)	< 0.001^***^	0.90 (0.56–1.44)	0.667
Severe	240	1.86 (1. 30–2.67)	< 0.001^***^	1.98 (1. 20–3.26)	0.007^**^
FIS-40					
Mild/moderate	512	1		1	
Severe	463	2.61 (2.01–3.39)	< 0.001^***^	1.27 (0.82–2.04)	0.311
D-FIS-8					
Lower fatigue	563	1		1	
Higher fatigue	514	2.44 (1.90–3.13)	< 0.001^***^	1.55 (1.08–2.21)	0.017^*^
PSQI					
Good sleepers	572	1		1	
Poor sleepers	446	1.47 (1. 14–1.90)	0.003^**^	0.98 (0.66–1.48)	0.913
SCL-90-R					
Mild/moderate	90	1		1	
No symptoms	125	1.1 (0.87–1.91)	0.217	1.30 (1. 15–2.73)	0.571
Severe	430	1.98 (1.51–2.61)	< 0.001^***^	1.34 (0.82–2.21)	0.243

*CI* confidence interval, *OR* odds ratio, *VAS* Visual analogue scale, *FIS* Fatigue impact scale, *SF-36* Short form 36-item health survey, *HADS* Hospital anxiety and depression scale, *PSQI* Pittsburgh sleep questionnaire index, *SCL-90-R* Symptom checklist-90-revised

^*^Significance at 0.05

^**^Significance at 0.01

^***^Significance at 0.001

^a^Data analysis using ANOVA

## Discussion

This is the first large community-based study in Spain to examine differential clinical assessment and fatigue-related variables related with work disability in CFS/ME patients who are unemployed at the time of diagnosis. More than half of patients were unemployed due to temporary or permanent disability. These results are consistent with those of Taylor’s review published in 2005 [23] which reported an unemployment rate of 35–69% and also with Ross’s study [24] which recorded a figure of 54% in CFS/ME patients: in a follow-up study in 2011, the latter authors found that the rate remained relatively stable at 50%. In a related illness like FM, a common comorbidity in CFS/ME subjects, the degree of unemployment ranges between 34 and 77% [25]. Patients over 50 at the time of diagnosis and those with a later onset of fatigue and pain have a higher risk of being unemployed. Collin et al. [26] also associated age with unemployment. In contrast to our study, those authors found lower percentage of women and higher proportions of patients ≤40 years; however, the percentage of patients who did not work was almost similar (58.6% in our study and 65.4% in Collin et al.’s study [26]).

The presence of a larger number of symptoms was indicative of unemployment in all clinical groups in the univariate analysis. However, in the multivariate model only autonomic dysfunction symptoms remained as a significant predictive factor. With regard to cognitive symptoms, more than 90% of our patients reported alterations in concentration, recent memory, and difficulty with reading and arithmetic, alterations which were more frequent in unemployed patients. These data coincide with a previous study by our group, which found also attention and information processing speed to be impaired in female patients with CFS/ME [27].

As for comorbidities, an interesting finding was the prevalence of FM: 62% in the unemployed compared with 46% of those in employment. In the univariate analysis, FM patients were 92% more likely to be unemployed than those without. A 2014 study by our team already showed that FM worsened clinical features, fatigue and QoL in CFS/ME patients [8]. Our data agree with those of Assefi et al. [28] who observed that the presence of FM increased unemployment (39% of CFS/ME patients but not FM were unemployed, compared with 66% of patients with both conditions). Other comorbidities such as degenerative vertebral disease and MCS were also associated with unemployment, in accordance with Brown et al.’s study [29].

In a 2005 review article, only depression was associated with occupational dysfunction in CFS/ME, although those authors concluded that longitudinal and intervention studies were necessary to confirm their data [23]. In the 2009 Hadlandsmyth study, depression was associated with work absenteeism and independently with fatigue in CFS/ME patients [30].

Our unemployed patients obtained higher scores on the fatigue perception scales. Higher scores on these scales emerged as a risk factor for unemployment in the univariate analysis, while in the multivariate analysis the association remained only for the VAS and the D-FIS-8. These data are in line with those of Collin et al. [26] and Knudsen et al. [31] Knudsen et al. found that patients with disability presented more fatigue than patients who remained in employment. Assessing fatigue with the Chalder scale, Collin et al. [26] found higher levels in unemployed patients, but in the multivariate analysis, fatigue was not maintained as a risk factor for work disability. In the study by Palstam et al. [32], fatigue was a symptom reported by FM patients; those in employment reported less physical and mental fatigue, as measured by the FIQ.

The use of the SF-36 questionnaire in CFS/ME has been previously described by several authors. In 2011, Nacul et al. [33] compared functional status and QoL in CFS/ME individuals and in other chronic illnesses such as depression, cancer and rheumatoid arthritis and found that the patients with the worst scores in the SF-36 were those diagnosed with CFS/ME. Other studies published by Jason et al. sought to identify the items on the SF-36 that best differentiate CFS/ME patients from healthy controls [34, 35]. Emotional role was the one with the poorest discrimination; with regard to the physical function, there were no significant differences in non-stressful activities such as bathing or walking at a slow pace, but differences emerged with more intense activity [31]. Nacul et al. suggested that the physical role item might be an adequate measure of outcome in CFS/ME, since it was the most affected [33]. Patients in our series had a poorer QoL (as measured by the SF-36 questionnaire) than CFS/ME patients from similar backgrounds reported in three previous studies [33, 35–37]. As a difference, scores on the items in Nacul et al.’s study [33] showed less variability than those of the other comparative studies, including ours. The confluence of the physical function score in all the studies (with the exception of Nacul et al. [33]) is also striking. However, the global physical and mental health scores were similar to those reported in the Nacul et al. study [33].

The only study that associates unemployment with QoL is the one by Collin et al. [26]; however, those authors measured only the physical function of the SF-36, which was identified as a risk factor in the multivariate analysis. In Palstam et al.’s study [32], patients with FM who were in work presented better physical health on the SF-36 scale.

Our results for the HADS scale were consistent with those of Collin et al. [26] Similarly, women with FM who worked obtained lower scores for depression on the HADS than those who did not, although no differences were observed for anxiety [32]. On the other hand, Ciccone [38] used the SCL-90-R as one of the variables to differentiate between patients who improved and those who did not. In the measurement of pain using the VAS, our results recall those of Collin et al. [26], who found higher VAS scores for pain in unemployed patients; these higher VAS scores were a risk factor for unemployment in the univariate model but not in the multivariate models. All participants had poor sleep quality as measured by the PSQI questionnaire. The unemployed obtained higher scores, indicating poorer sleep quality. Reviewing the literature, no previous studies have assessed work-related incapacity and sleep quality in CFS/ME individuals.

This study has several limitations and strengths. The first limitation that we should stress is the fact that the patients were all recruited from a tertiary-referral hospital unit for CFS/ME. Most were referred from primary care, but others were referred from a specialist care unit, and so the population as a whole might be expected to present more severe symptoms than if the study had been carried out exclusively in the primary care setting. Second, as this was a cross-sectional study, the evolution of patients’ employment status over time could not be assessed. Third, in unemployed patients the reason for temporary or permanent work incapacity was unknown (no distinction was made between full-time and part time employment). Fourth, it is difficult to judge the significance of these findings as no attempt was made to compare these results with those of matched healthy controls. One of the main strengths of this study was the wide-cohort of CFS/ME patients who were included prospectively, which allowed us to evaluate all the predictive variables under adequate data analysis. All patients recruited had CFS/ME diagnosed by a specialist; patients with fatigue secondary to other health conditions were excluded. In the assessment of work disability, individuals whose employment status could not be assessed at the time of diagnosis were also excluded.

## Conclusions

In summary, our findings suggest that the CFS/ME phenotype that predicts of work disability at the time of diagnosis in a tertiary hospital CFS/ME clinical setting is that of a woman aged over 50 years, with professional qualifications and a medium or high educational level, with notable neurocognitive symptoms, associated comorbidities, high levels of fatigue, pain, psychopathology symptoms, sleep problems, and poor physical and mental quality of life. These data may help to guide optimal functional assessment and rehabilitation therapy in unemployed CFS/ME patients. Some possible interventions and/or strategies at macro- or policy, meso- and micro-levels frame in populations of CFS/ME sufferers might be: 1) an adaptation of the working conditions (hours worked, physical and cognitive effort, etc) could be proposed - this adaptation should be personalized to the needs of each patient with health care personnel trained to manage the illness supported by government policies around the world; 2) provide evidence-based treatments with demonstrated efficacy and safety useful in individuals who has CFS/ME, not treating the illness; and then 3) promote cooperation programmes providing counseling, education, information feedback, and other supports to patients in CFS/ME clinical units from health care system in order to improve the outcomes both in Spain and abroad. Further additional studies are now needed to focus on work-related disability in CFS/ME in order to develop and support potential rehabilitation strategies for this condition.

## Data Availability

Not applicable. The datasets generated during the study are not publicly available due to an ethical restriction (patient confidentiality) but are available from the corresponding author on reasonable request.

## Abbreviations

ACAF: Association of Central Sensitization Syndrome Patients from Catalonia, Spain
CDC: Centers for Disease Control and Prevention
CFS/ME: Chronic Fatigue Syndrome/Myalgic Encephalomyelitis
D-FIS: Daily Fatigue Impact Scale
FDA: Food and Drug Administration
FIQ: Fibromyalgia Impact Questionnaire
FIS: Fatigue Impact Scale
FM: Fibromyalgia
HADS: Hospital Anxiety and Depression Scale
ISCED: International Standard Classification of Education
MCS: Multiple Chemical Sensitivity
PSQI: Pittsburg Sleep Quality Index
QoL: Quality of Life
SCL-90R: Symptom ChecList-90-Revised
SF-36: Short Form 36-item Health Survey
SNHS: Spanish National Health System
VAS: Visual Analog Scale
